# Risk of Prevalent Asthma among Children Affected by Inflammatory Bowel Disease: A Population-Based Birth Cohort Study

**DOI:** 10.3390/ijerph17124255

**Published:** 2020-06-15

**Authors:** Claudio Barbiellini Amidei, Fabiana Zingone, Loris Zanier, Cristina Canova

**Affiliations:** 1 Unit of biostatistics, Epidemiology and Public Health. Department of Cardio-Thoraco-Vascular Sciences and Public Health, University of Padua, 35100 Padua, Italy; claudioamidei@gmail.com; 2 Department of Surgery, Oncology and Gastroenterology, Gastroenterology Section, University Hospital of Padua, 35100 Padua, Italy; fabiana.zingone@unipd.it; 3 Epidemiological Service, Health Directorate, 33100 Udine, Italy; loris.zanier110@gmail.com

**Keywords:** inflammatory bowel disease, childhood onset IBD, pediatric IBD, VEO-IBD, EO-IBD, asthma, birth cohort study, postnatal exposure, real-world data, record linkage

## Abstract

Literature on the risk of asthma among children with inflammatory bowel disease (IBD) is limited and has reported discording results. To the best of our knowledge, no previous study has evaluated the association between asthma and childhood onset IBD, focusing on pediatric IBD with onset between 10 and 17 years, early-onset IBD (EO-IBD) between 0 and 9 years, and very early-onset IBD (VEO-IBD) between 0 and 5 years, all conditions characterized by different clinical progressions. A nested matched case-control design on a longitudinal cohort of 213,515 newborns was adopted. Conditional binomial regression models were used to estimate odds ratios (OR) and 95% confidence intervals (CI) of asthma among children with IBD compared with controls. We found 162 children with IBD and 1620 controls. Overall, childhood onset IBD was associated with increased risks of being affected by asthma (OR: 1.49 95% CI 1.05–2.12), although a significant risk was only present among males (OR: 1.60 95% CI 1.02–2.51). Children with Crohn’s disease and ulcerative colitis had similarly increased risks, although they failed to attain statistical significance. Risks of asthma based on age at IBD onset were inversely related to age, with the lowest non-significant risks for pediatric IBD and EO-IBD, while children affected by VEO-IBD had the highest risk of asthma (OR: 2.75 95% CI 1.26–6.02). Our study suggests the presence of a higher prevalence of asthma among both male children with IBD and children with VEO-IBD. It could be advisable to pay greater attention to possible respiratory symptoms among these categories at higher risk.

## 1. Introduction

Inflammatory bowel disease (IBD) and asthma are two relevant and very impactful immune-mediated diseases that share common genetic and environmental risk factors [1].

IBD consists of a group of relapsing conditions that includes Crohn’s disease and ulcerative colitis, characterized by chronic inflammation of the gastrointestinal tract, a progressive course, and potential development of complications including extra-intestinal manifestations. IBD can occur at any age, with the highest incidence between 15 and 29 years [2,3]. Childhood onset IBD (defined as IBD before 18 years) is increasing especially in Western countries, with a stable incidence before 6 years of age, but an increasing trend between 6 and 16 years [4,5]. Gender-based differences seem to vary according to the type of IBD, as recently shown by a pooled analysis on the prevalence of IBD in Western countries, according to which more male children are affected by Crohn’s disease among patients aged 0 to 14 years, while slightly more female subjects are affected by ulcerative colitis among those aged 0 to 9 years [6]. Increasing interest has grown for a different clinical progression of childhood onset IBD, based on the age at disease onset, categorized according to the latest Paris classification in pediatric IBD between 10 and 17 years, and early-onset IBD (EO-IBD) between 0 and 9 years [7]. Another group of patients characterized by distinct clinical features are children with very early-onset IBD (VEO-IBD) between 0 and 5 years [8,9].

Asthma is a common pediatric disease, characterized by chronic inflammation of the respiratory tract [10,11], with an increasing prevalence in Western countries, similarly to other immune-mediated conditions [12,13]. The incidence of asthma has a peak in early childhood and then progressively decreases with age [10,14]. Gender prevalence differences of childhood asthma are well established, with higher risks among males (2:1 ratio) until the beginning of puberty, when relevant hormonal changes even out sex-based differences (1:1 ratio) [15,16,17].

Some studies have observed an association between IBD and asthma [18,19,20,21,22], but others have reported contrasting results or have confirmed this association only for specific types of IBD [20,23,24,25,26]. A recent meta-analysis by Kuenzig et al. has shown increased risks for co-occurrence of asthma for both Crohn’s disease and ulcerative colitis, when considering the entire population. Nevertheless, no significant association has been observed in the pediatric population, neither for Crohn’s disease nor ulcerative colitis [1].

IBD and asthma share an abnormal immune response to antigenic stimuli and a common barrier dysfunction of the epithelial layer, a pathological feature that can concern both the gastrointestinal tract and the respiratory system [27,28,29,30,31]. Several biological explanations have been proposed for the association between these two diseases. Among these are possible alterations of the microbiome [32,33], the ‘hygiene hypothesis’ according to which an increasing prevalence of immune-mediated diseases would be linked to a reduction of antigenic stimuli deriving from pathogens and environmental exposures during early childhood [34], as well as explanations linked to genetic susceptibility [35] and alterations of physiological inflammatory processes [36,37].

To the best of our knowledge, past studies have not thoroughly investigated differences in the risk of co-occurrence of childhood onset IBD and asthma, especially in relation to gender and specific age at IBD onset. Furthermore, results in the literature regarding this association are discordant; therefore, the aim of this study is to analyze the risk of prevalent asthma among children affected by IBD in a population-based birth cohort, stratifying by sex, type of IBD (Crohn’s disease and ulcerative colitis), and age at IBD onset, analyzing VEO-IBD separately.

## 2. Methods

### 2.1. Study Population

The study population consisted of all children born between 1989 and 2012 in the region of Friuli-Venezia Giulia (North-eastern Italy). This region has 1.2 million residents with about 10,000 newborns per year. A regional integrated healthcare system developed in the 1980s allows to automatically collect and pool data on all healthcare services provided to every resident by the National Health Service, by means of a unique regional identification code. A more detailed explanation of the system’s functioning and the data sources available has been reported in a previous paper [38]. Healthcare administrative databases included in this study were as follows: hospital discharge records (with up to six diagnoses coded according to the International Classification of Diseases Ninth Revision Clinical Modification (ICD-9-CM) for each hospitalization), drug prescription records database (coded according to the Anatomical Therapeutic Chemical (ATC) classification system, available from 1995 onward), healthcare co-payment exemptions database (recorded with a national coding system), and mortality records. Every new-born in the birth cohort, held in the medical birth register, was linked through their unique regional identification code to each of these healthcare administrative databases. The information recorded in the medical birth register for every delivery in the region includes socio-demographic data regarding the parents, pregnancy, labor, delivery, and the newborn at birth.

### 2.2. IBD Definition

Children with IBD onset before the age of 18 years were defined by the presence of at least one of the following: a hospital discharge record reporting in any of the six possible diagnoses the ICD-9-CM codes: 555 (regional enteritis), 556 (ulcerative colitis) (excluding ICD-9-CM codes: 556.0 chronic ulcerative enterocolitis, 556.1 chronic ulcerative ileocolitis, 556.4 colonic pseudopolyposis, 556.8 other ulcerative colitis), or an exemption for healthcare co-payment (national coding system: 009.555 for both Crohn’s disease and ulcerative colitis). This case-identification algorithm has been validated in a previously published paper, with a reported sensitivity of 75.4% exclusively for hospital discharge records and 82.2% for hospitalizations and healthcare co-payment exemptions combined [39]. IBD onset date was defined as the earliest date among the two sources used (hospital discharge records and healthcare co-payment exemptions).

On the basis of the age at IBD diagnosis, childhood onset IBD was categorized in three groups: pediatric IBD between 10 and 17 years (Paris classification A1b), EO-IBD between 0 and 9 years (Paris classification A1a), and VEO-IBD between 0 and 5 years [8,40].

### 2.3. Asthma Definition

Children with current asthma (from here on labeled as “asthma”) after the age of 3 years were identified by means of the drug prescription record database. Asthma was defined by the presence in a 12-month window of at least two prescriptions among the following: short- and long-acting beta2 agonists (ATC code R03AC*), adrenergics in combination with other drugs (not anticholinergics) (ATC code R03AK*), inhaled corticosteroids (ATC code R03BA*), and antileukotriene drugs (ATC code R03DC*). Children that matched this definition before the age of 3 years were not excluded from the analyses, but were considered asthmatics only if they matched the algorithm’s inclusion criteria also after 3 years of age. This algorithm was based on a validated one used to estimate asthma prevalence, with a positive predictive value of 78.5% (95% CI 76.2–80.7) and a sensitivity of 74.5% (95% CI 72.1–76.7) [41].

### 2.4. Statistical Analyses

A nested matched case-control study design was adopted. Through a macro iterative process of the Statistical Analysis System (SAS), all IBD cases were identified from the cohort. All possible controls were identified for each case among newborns in the same population, recorded in the medical birth register. Controls were matched by sex and year of birth, were alive at 1 year of age (after merging with mortality records), and resided in Friuli-Venezia Giulia at the moment of IBD diagnosis. Among all possible controls, ten children were randomly selected for each case. An index date, corresponding to the date of IBD diagnosis of their matched case, was assigned to every control. All analyses were restricted to subjects with index date from 1995 onward (year from which drug prescription records are available).

Conditional binomial regression models were used to estimate the odds ratio (OR) with 95% confidence interval (CI) for asthma among children affected by IBD compared with references within the same stratum. Age and sex confounding were thus always controlled by the study design. In our analyses, we included all diagnoses of asthma regardless if they preceded or followed the diagnosis of IBD.

Analyses were stratified by sex, type of IBD (Crohn’s disease and ulcerative colitis), and age at IBD onset (pediatric IBD, EO-IBD, and VEO-IBD). Stratified analyses for Crohn’s disease and ulcerative colitis were restricted to children identified by means of hospital discharge records, as healthcare co-payment exemptions reported the same code, and it was not possible to distinguish between these two conditions. Children with both a diagnosis of Crohn’s disease and ulcerative colitis in two separate hospital discharge records were excluded from the stratified analyses.

A sensitivity analysis was performed, considering asthma onset at age 6 or older as secondary outcome, to increase the specificity of the diagnosis. This sensitivity analysis restricted the population to children who were alive and resided in the region at the age of 6 years. A further sensitivity analysis (restricted to children born from 1995 onward) was performed by adjusting for antibiotic exposure (as categorical variable) in the first year of life.

All information present in the databases we used was completely anonymized and de-identified prior to analyses. Therefore, no informed consent and no ethics committee approval was required.

Statistical analyses were conducted with SAS software v 9.4 (SAS Institute, Cary, NC, USA).

## 3. Results

The birth cohort consisted of 213,515 children, born and residing in Friuli-Venezia Giulia, North-eastern Italy. By means of hospital discharge records and healthcare co-payment exemptions, we identified 162 subjects with childhood onset IBD and 1620 controls, matched by sex and year of birth. The exclusive contribution of hospital discharge records to identify children affected by IBD was 40% and that of healthcare co-payment exemptions was 6%, while these two sources combined lead to identifying 54% of cases. On the basis of hospital discharge records, it was possible to establish, for 144 children, whether they were affected by Crohn’s disease (81 cases, 56.2%) or ulcerative colitis (63 cases, 43.8%).

Overall, the IBD population was composed of 94 males (58%) and 102 youths (63%) diagnosed with pediatric IBD (10–17 years). The main characteristics of individuals affected by IBD and their controls are reported in Table 1.

The risk of being affected by asthma was higher among subjects with any type of IBD compared with references (OR: 1.49 95% CI 1.05–2.12), with a significantly increased risk only for males (OR: 1.60 95% CI 1.02–2.51), as shown in Table 2.

Among the 56 children with IBD and asthma, 47 (83.9%) had been diagnosed with asthma before IBD onset. Prevalence of asthma appeared to be increased in both children affected by Crohn’s disease (OR: 1.29 95% CI 0.78–2.14) and ulcerative colitis (OR: 1.59 95% CI 0.92–2.74), compared with references, although the results failed to attain statistical significance. When stratifying by age, younger children with IBD had a higher risk of asthma. Children with VEO-IBD in fact had the highest risks (OR: 2.75 95% CI 1.26–6.02), and these estimates lowered progressively without reaching statistical significance) for EO-IBD (OR: 1.70 95% CI 0.95–3.05) and pediatric IBD (OR: 1.39 95% CI 0.90–2.15), as shown in Table 3.

Analyses for the risk of asthma at the age of 6 years were also performed (Supplementary Table S1). Risks were similar to those observed in the main analyses, but failed to attain statistical significance due to low numerosity.

Given recent evidence suggesting that antibiotic exposure in the first year of life is associated with both IBD [9,40] and asthma [42,43], a sensitivity analysis adjusted for this exposure was performed on 70 IBD cases and 700 references (analyses restricted to children born after 1994). Antibiotic exposure in the first year of life showed a non-significant increased risk of asthma at 3 years (OR 1.62 95% CI 0.91–2.69).

## 4. Discussion

This paper suggests that children affected by IBD are at increased risk of being affected by asthma. Specifically, this risk appears to be higher among male children with IBD and children with VEO-IBD. Asthma diagnosis in 47 out of 56 children (83.9%) preceded IBD diagnosis, and this could be explainable by an especially high prevalence of asthma in early childhood [10]. To the best of our knowledge, this is the first study to analyze the comorbidity of asthma among children affected by IBD, focusing on specific subgroups defined by age at IBD onset, categorized according to the latest Paris classification and with a further distinction regarding children with VEO-IBD.

When analyzing all types of IBD, gender-based differences highlighted how risks were concentrated among male children. Conversely, previous literature has observed an association between asthma and IBD especially among female subjects, but these studies did not focus on childhood onset IBD [23,44]. Brassard et al. have in fact noticed how a higher incidence of Crohn’s disease was present among women affected by asthma after 10 years of age, compared with asthmatic men [23]. The reasons as to why age appears to modify these sex-based differences are not clear and will require further investigation. However, hormones have already been shown to play a role in IBD exacerbations during pregnancy [45] and are responsible of sex-prevalence differences in asthma [16,17]. Studies that will evaluate the role of hormones in the pathophysiology of childhood onset IBD could be of great use to better understand the mechanisms that underlie these differences.

Our analyses for Crohn’s disease and ulcerative colitis have shown non-significantly increased risks of asthma. The results from past literature on risks associated to these two types of IBD are controversial. Raj et al., for instance, found an association only between ulcerative colitis and asthma (OR 2.81 95% CI 1.15–6.9) [26]. On the other hand, two other studies observed significantly increased risks (ranging from 1.42 to 2.33) only for the development Crohn’s disease [20,24].

Stratified analyses by age at IBD onset have shown how the risk of asthma diminished with the increase of IBD onset age. VEO-IBD was in fact associated with the highest risks of asthma (OR: 2.75 95% CI 1.26–6.02), while EO-IBD (OR: 1.70 95% CI 0.95–3.05) and pediatric IBD (OR: 1.39 95% CI 0.90–2.15), although they failed to attain statistical significance, were associated with progressively lower risks. In a paper by Brassard et al., stratified analyses by type of IBD and age at IBD onset showed an increased incidence rate ratio of Crohn’s disease among asthmatic children aged 0 to 9 years (2.11 95% CI 1.7–2.61), but found no association among those aged 10 to –19 years, for neither Crohn’s disease nor ulcerative colitis [23]. Another study, which had a similar stratification, but with different age cut-offs, observed increased risks among asthmatic subjects aged ≤16 years, only for the development of ulcerative colitis (OR: 1.49 95% CI 1.08–2.07) [25]. Similar to what we observed, pooled data from a meta-analysis have shown no significantly increased risks for pediatric onset (defined as ≤16 years) Crohn’s disease (pooled relative risks 1.35 95% CI 0.94–1.93) or pediatric onset ulcerative colitis (pooled relative risks 1.11 95% CI 0.97–1.28), despite point estimates being slightly elevated [1]. However, a difference between this meta-analysis and our study regarded the cut-off applied for age stratification, which was based on the Montreal classification, which defines pediatric onset IBD as ≤16 years, unlike the more recent Paris classification that distinguishes between pediatric IBD (10–17 years) and EO-IBD (0–9 years) [1,7], and the meta-analysis also did not consider VEO-IBD (0–5 years). The broader cut-offs in the Montreal classification could in fact mask the increased risks among more susceptible groups within this heterogeneous category, as suggested by our results. This is partly supported by the findings of Brassard et al., who observed increased risks among asthmatic subjects, exclusively for the development of Crohn’s disease, between 0 and 9 years, but no risks between 10 and 19 years [23]. Despite analyzing for Crohn’s disease and ulcerative colitis separately, these age-stratified results could reinforce the growing evidence that suggests an actual difference in the clinical characteristics of IBD based on age at onset, especially for VEO-IBD [8,9]. It has been hypothesized, in fact, that VEO-IBD may represent a phenotypically different type of IBD, as opposed to later onset IBD [8]. Children with VEO-IBD have been receiving increasing attention by the scientific community for specific genetic characteristics and different health trajectories that characterize the clinical progression of this disease [8,9,46].

From a pathophysiological perspective, IBD and asthma are both characterized by an excessive immune response to antigenic stimuli. A relevant role is played by a dysfunction of epithelial layers, which may concern both the gastrointestinal tract as well as the respiratory tract [27,28,29,30,31]. In asthmatic individuals, the respiratory tract is characterized by a damaged or abnormal epithelial layer that does not perform a proper barrier function, thereby enhancing mucosal permeability to foreign substances in the airway epithelium, with a subsequent increase in the release of epithelial cytokines and the promotion of an inflammatory process [27]. A disrupted integrity of the mucosal cell lining in the bowel plays a similar role in IBD. This results in an ineffective physical barrier, but also, due to a lack of defensive pattern recognition receptors, in a possible promotion of chronic inflammation [31].

Several other hypotheses have been proposed to explain the association between IBD and asthma. One of them relates to an alteration of the gastrointestinal microbiome. Dysbiosis can occur for several reasons, among which are breast-feeding, dietary changes, lifestyle changes, and exposure to specific medications such as antibiotics, as well as, according to recent evidence, acid-suppressive medications [32,33,42,47]. Modifications of the human microbiome have been associated with altered immune responses that lead to higher risks of developing immune-mediated disorders [48,49], while a healthy microbiome is linked to an increased presence of regulatory T-cells that contribute to modulating immune responses [50]. Animal models suggest that alterations of the microbiome seem to be especially impactful early in life [51]. Evidence in humans also suggests that dysbiosis may play a relevant role in both the development of IBD [52] and asthma [53].

Another hypothesis, the ‘hygiene hypothesis’, would link the recent reduction of antigenic stimuli (partly deriving from early-life infections, partly from environmental exposures) during infancy and early childhood to the development of immune-mediated diseases [34]. This hypothesis would be especially relevant in Western countries, where hygienic conditions underwent a constant improvement [54]. Anyway, what has been observed is an increase in the prevalence of IBD and asthma, as well as other immune-mediated conditions [4,5,12,13,34].

IBD and asthma also share a common genetic susceptibility, which includes a polymorphism of gasdermin-B (GSDMB), a protein responsible for regulating differentiation and growth of epithelial cells [35], as well as a polymorphism for the Interleukine 23 receptor gene [36]. Furthermore, these two diseases seem to have a common therapeutic target, TL1A (part of the superfamily of tumor necrosis factor ligands), that, when inhibited by an anti-TL1A antibody, induces a reduction of the mucosal inflammation [37].

A strength of our study is the presence of a population-based design, characterized by a long follow-up with an automatic registration of data regarding all healthcare services delivered by the National Health Service, which avoids any possible recall bias. Individuals with childhood onset IBD were in fact identified by linkage with both hospital discharge records and a disease-specific healthcare co-payment exemption database. This way, we believe to have captured an elevated proportion of cases, without being impacted by biases related to the frequency or severity of healthcare service utilization. This validated case-identification algorithm was in fact defined to maximize sensitivity by identifying children in both an inpatient healthcare setting (hospital discharge records) and an outpatient setting (healthcare co-payment exemption). Healthcare co-payment exemptions are provided by doctors directly at the moment of IBD diagnosis, and are thus easily obtainable from all subjects affected by this condition. Nevertheless, a limit of this algorithm is linked to the months of delay between the actual beginning of the inflammatory process and the onset of symptoms that then lead to a medical diagnosis [55]. However, owing to the longitudinal study design and the inclusion of children with asthma onset before and after IBD diagnosis, we believe this limit should not have impacted our results.

Another possible limitation of our study is that we could not directly assess the presence of asthma; therefore, all cases have been identified by evaluating anti-asthmatic drug prescriptions. Nevertheless, the algorithm we applied has been validated in a previous study that reported a positive predictive value of 78.5% (95% CI 76.2–80.7) and a sensitivity of 74.5% (95% CI 72.1–76.7) [41]. A possible bias we could not rule out is related to the fact that children affected by asthma could receive closer medical attention and be diagnosed with IBD more promptly, compared with non-asthmatic children. Nevertheless, we believe this bias is not sufficient to explain the very elevated risks found among children with VEO-IBD and the slightly increased risks observed among male children with IBD.

## 5. Conclusions

This study has found an association between asthma and childhood onset IBD. This excess risk was especially concentrated among male children with IBD and children with VEO-IBD. On the basis of our results, it may be worth providing specific attention for respiratory symptoms to these categories at increased risk. This multidisciplinary approach could help to assure a better healthcare support for these patients. Nevertheless, further studies are needed to confirm our findings as well as studies to assess what exact pathophysiological mechanisms underlie these observations.

## Figures and Tables

**Table 1:** Distribution by Sex, Year of Birth, Type of Inflammatory Bowel Disease (IBD), and Age at IBD onset among Cases and Controls.

Study Population Characteristics	Cases (N = 162) n (%)	Controls (N = 1620) n (%)
Sex		
Male	94 (58.0%)	940 (58.0%)
Female	68 (42.0%)	680 (42.0%)
Calendar year of birth		
1989–1993	65 (40.1%)	650 (40.1%)
1994–1998	57 (35.2%)	570 (35.2%)
1999–2004	23 (14.2%)	230 (14.2%)
2005–2012	17 (10.5%)	170 (10.5%)
Type of IBD		
Crohn’s disease ^1^	81 (56.2%)	
Ulcerative colitis ^1^	63 (43.8%)	
Pediatric IBD ^2^	102 (63.0%)	
EO-IBD ^3^	60 (37.0%)	
VEO-IBD ^4^	33 (20.4%)	

^1^ Only among 144 children with a hospital discharge record diagnosis of Crohn’s disease or ulcerative colitis; ^2^ pediatric IBD: age at IBD onset between 10 and 17 years; ^3^ early-onset IBD (EO-IBD): age at IBD onset between 0 and 9 years; ^4^ very early-onset IBD (VEO-IBD): age at IBD onset between 0 and 5 years.

**Table 2:** Risk of Asthma among Children Affected by IBD, Stratified by Sex. OR, Odds Ratio; CI, Confidence Interval.

Sex	Population at Risk	Asthmatic Subjects	%	OR (95% CI)
All	Any type of childhood onset IBD (N = 162)	56	34.6	1.49 (1.05–2.12)
	References (N = 1620)	427	26.4	1
Male	Any type of childhood onset IBD (N = 94)	35	37.2	1.60 (1.02–2.51)
	References (N = 940)	258	27.4	1
Female	Any type of childhood onset IBD (N = 68)	21	30.9	1.36 (0.79–2.34)
	References (N = 680)	169	24.9	1

**Table 3:** Risk of Asthma among Children Affected by IBD, Stratified by Type of IBD and Age at IBD onset.

Population at Risk	Asthmatic Subjects	%	OR (95% CI)
Type of IBD			
Crohn’s disease ^1^ (N = 81)	25	30.9	1.29 (0.78–2.14)
References (N = 810)	210	25.9	1
Ulcerative colitis ^1^ (N = 63)	23	36.5	1.59 (0.92–2.74)
References (N = 630)	168	26.7	1
Age at IBD onset			
Pediatric IBD ^2^ (N = 102)	36	35.3	1.39 (0.90–2.15)
References (N = 1020)	289	28.3	1
EO-IBD ^3^ (N = 60)	20	33.3	1.70 (0.95–3.05)
References (N = 600)	138	23.0	1
VEO-IBD ^4^ (N = 33)	13	39.4	2.75 (1.26–6.02)
References (N = 330)	66	20.0	1

^1^ Only among 144 children with a hospital discharge record diagnosis of Crohn’s disease or ulcerative colitis; ^2^ pediatric IBD: age at IBD onset between 10 and 17 years; ^3^ early-onset IBD: age at IBD onset between 0 and 9 years; ^4^ very early-onset IBD: age at IBD onset between 0 and 5 years.

## Supplementary Materials

The following are available online at https://www.mdpi.com/1660-4601/17/12/4255/s1, Table S1: Sensitivity analysis of the risk of asthma at 6 years, among children affected by IBD.
