# Supplementary material for: Risk of Prevalent Asthma among Children Affected by Inflammatory Bowel Disease: A Population-Based Birth Cohort Study
Source: Int J Environ Res Public Health. 2020 Jun 15;17(12):4255. doi: 10.3390/ijerph17124255 (PMC7345598; doi:10.3390/ijerph17124255)
Supplement: Supplementary file 1 [file ijerph-17-04255-s001.pdf]

International Journal of
Environmental Research and Public Health

MDPI

Supplementary Table S1 Sensitivity Analysis of the Risk of Asthma at 6 Years, among Children Affected by IBD.

Population at Risk	Asthmatic Subjects	%	OR (95% CI)
All IBD			
All subjects (N=162)	34	21.0	1.42 (0.95-2.13)
References (N=1620)	257	15.9	1
Male (N=94)	23	24.5	1.52 (0.92-2.50)
References (N=940))	166	17.7	1
Female (N=68)	11	16.2	1.26 (0.63-2.50)
References (N=680)	91	13.4	1
Type of IBD			
Crohn's Disease [1] (N=81)	15	18.5	1.24 (0.68-2.26)
References (N=810)	126	15.6	1
Ulcerative Colitis [1] (N=63)	14	22.2	1.47 (0.78-2.78)
References (N=630)	103	16.4	1
Age at IBD Diagnosis			
Pediatric IBD [2] (N=102)	22	21.6	1.25 (0.76-2.05)
References (N=1020)	184	21.6	1
EO-IBD [3] (N=60)	12	20.0	1.88 (0.93-3.80)
References (N=600)	73	12.2	1
VEO-IBD [4] (N=33)	6	18.2	2.68 (0.96-7.44)
References (N=330)	27	8.2	1

[1] Only among 144 children with a hospital discharge record diagnosis of Crohn's disease or ulcerative colitis; [2] Pediatric IBD: age at IBD diagnosis between 10 and 17 years; [3] Early-Onset IBD: age at IBD diagnosis between 0 and 9 years; [4] Very Early-Onset IBD: age at IBD diagnosis between 0 and 5 years.
